# Geographic and Behavioral Determinants of Typhoid and Antimicrobial Resistance in Children Across Urban, Rural, and Nomadic Populations of Punjab, Pakistan

**DOI:** 10.3390/healthcare14010124

**Published:** 2026-01-04

**Authors:** Atifa Ambreen, Muhammad Asif Zahoor, Muhammad Hidayat Rasool, Mohsin Khurshid

**Affiliations:** Institute of Microbiology, Government College University Faisalabad, Faisalabad 38000, Pakistan; atifa.ambreen@gmail.com (A.A.); drmhrasool@gcuf.edu.pk (M.H.R.); mohsinkhurshid@gcuf.edu.pk (M.K.)

**Keywords:** *Salmonella* Typhi, surveillance, typhoid fever, XDR, risk factors

## Abstract

**Highlights:**

**What are the main findings?**

- A high burden of typhoid (41.5% proportion of culture-positive cases) among children in Punjab, especially affecting nomadic and low-income populations.
- Extensive antimicrobial resistance is prevalent, with 88.8% of isolates resistant to ciprofloxacin and 83.7% and 73.8% to trimethoprim-sulfamethoxazole and ampicillin, respectively, which was strongly linked to the presence of *sul1*, *sul2*, *dfrA7*, and *bla*_TEM_ genes.

**What are the implications of the main findings?**

- The findings highlight the urgent need for expanded typhoid conjugate vaccine (TCV) coverage, improved water, sanitation, and hygiene (WASH) infrastructure, and antibiotic stewardship in high-burden communities.
- Integrating behavioral education with molecular surveillance is critical for controlling typhoid transmission and monitoring emerging antimicrobial resistance in Pakistan.

**Abstract:**

**Background/Objectives**: This study aimed to determine the antimicrobial susceptibility patterns of multidrug resistant (MDR) and extensively resistant (XDR) *Salmonella enterica serovar* Typhi (*S*. Typhi) strains among children, along with the associated behavioral and environmental risk factors across different population groups in multiple districts of Punjab, Pakistan. **Methods**: A cross-sectional study was conducted across 20 districts in Punjab, Pakistan. Structured questionnaires were used to assess sociodemographic and behavioral determinants. Blood cultures from febrile children were obtained for the isolation and identification of *S*. Typhi, followed by antimicrobial susceptibility testing and screening for the resistance genes. **Results**: A total of 900 blood samples were collected and 41.5% were positive for *S*. Typhi. The proportion of culture-positive cases were higher among children aged 6–12 years (34.8%). Sociodemographic and behavioral analysis revealed that children from low-income households (PKR < 20,000 showed significantly higher infection rate (67.1%, *p* < 0.001). Antimicrobial susceptibility testing revealed high resistance rates against several antibiotics: Ciprofloxacin (88.8%), Trimethoprim/sulfamethoxazole (83.7%), Ampicillin (73.8%) and Chloramphenicol (72.7%). However, all isolates remained susceptible to carbapenems and azithromycin. The prevalence of MDR and XDR *S.* Typhi in urban areas was 28.1% and 60.8%, respectively, while rural areas showed 22.6% MDR and 20.6% XDR. In contrast, nomadic populations exhibited a higher rate of MDR (49.3%) but a lower XDR prevalence of 18.6% with significant geographic variations in resistance patterns. Molecular analysis revealed a high prevalence of resistance genes, including *sul1* (83.7%), *sul2* (79.7%), followed by *dfrA7* (81.3%), *catA1* (64.9%) and *bla*_TEM_ (60.5%), *bla*_CTX-M-1_ (12.5%), *bla*_CTX-M-15_ (25.9%) and *qnrS* (88.8%), respectively. **Conclusions**: The study underscores a persistent typhoid burden and widespread antimicrobial resistance among children in Punjab. Targeted vaccination, antibiotic stewardship, public health education are urgently needed, especially among the nomadic population, where healthcare access and hygiene awareness are limited.

## 1. Introduction

Typhoid fever, caused by *Salmonella enterica* serovar Typhi (*S.* Typhi), remains a severe global health threat, predominantly in low- and middle-income countries (LMICs) where inadequate access to safe water, sanitation, and hygiene (WASH) fuels transmission [1]. The global burden is substantial, with an estimated 13.5 million cases and 135,000 deaths annually, corresponding to the incidence rate of 2.14 per 1000 people [2]. The latest WHO fact sheet (as of 2023) reports approximately 9 million cases and 110,000 deaths per year based on 2019 modeling. More recent analyses, including the Global Burden of Disease (GBD) study, indicate further declines: 9.2 million cases (95% CI: 5.9–14.1 million) and 110,000 deaths (95% CI: 53,000–191,000) in 2019, with GBD estimating over 7 million cases and more than 93,000 deaths in 2021 [3]. This disparity is starkly evident in Pakistan, where typhoid is a leading cause of mortality and a major bacterial cause of febrile illness; the incidence among children aged 2 to 5 years is particularly high, at 573.2 per 100,000 per year [4].

Transmission occurs primarily via the fecal-oral route through two pathways: direct contact with contaminated food and water due to poor hygiene, street food consumption, and inadequate sanitation; and indirect exposure from environmentally contaminated sources, such as sewage-polluted water supplies or the use of untreated waste in agriculture [5]. Rapid urbanization is shifting the epidemiological landscape, with urban centers now experiencing higher transmission rates than rural areas [6]. In Pakistan, a country with diverse population groups including urban, rural, and nomadic communities, the risk is amplified by a confluence of factors including overcrowding, water contamination, antibiotic misuse, and limited healthcare access [7]. The unique challenges faced by rural and nomadic communities, which are normally characterized by lower educational attainment and income and are frequently overlooked, exacerbating these health disparities [8].

A critical complication in typhoid management is the rapid emergence of antimicrobial resistance (AMR). Multidrug-resistant (MDR) *S.* Typhi strains, resistant to first-line antibiotics like ampicillin, chloramphenicol, and trimethoprim-sulfamethoxazole, are now widespread [9,10,11]. This has necessitated a shift to second- and third-line antibiotics, including fluoroquinolones and cephalosporins [12]. Alarmingly, extensively drug-resistant (XDR) *S.* Typhi strains, resistant to these additional drug classes, have since emerged. A major outbreak was first reported in Pakistan’s Sindh province in 2016 [9], leading to thousands of cases and international spread, underscoring the gravity of the XDR threat [13]. In response, the WHO has prioritized *S.* Typhi due to its escalating resistance and high associated morbidity, particularly in children from LMICs [14].

Despite these escalating challenges, comprehensive data on the geographic heterogeneity of typhoid burden, associated risk factors, and local AMR patterns across different population groups in Pakistan are lacking. To address this critical knowledge gap, we conducted extensive surveillance across 20 districts in Punjab, Pakistan. Our study integrates detailed questionnaire data on behavioral and environmental risk factors with blood culture confirmation from febrile patients. We aimed to determine the proportion of clinically suspected febrile children with culture-confirmed *Salmonella* Typhi, characterize the contemporary antimicrobial resistance profile, and conduct a comprehensive genetic analysis of resistance mechanisms to inform targeted public health strategies.

## 2. Materials and Methods

### 2.1. Study Design and Sampling

This descriptive cross-sectional study was conducted from April 2024 to August 2025 in Punjab, Pakistan. The study employed a stratified, non-random, facility-linked sampling approach, with stratification by place of residence (urban, rural, and nomadic populations). A total of 900 febrile children (≤15 years) were enrolled, with equal target numbers of 300 children per population group. This sample size was selected to ensure balanced representation across population strata, facilitate meaningful comparisons between urban, rural, and nomadic settings, and remain feasible within logistical and resource constraints across multiple districts.

Participants were recruited from healthcare facilities and outreach services (including outpatient departments, emergency units, and rural health centers) located within 20 selected districts of Punjab including Lahore (LHR), Faisalabad (FSB), Sheikhupura (SKP), Gujranwala (GRW), Narowal (NWL), Sargodha (SGD), Sahiwal (SWL), Gujrat (GRT), Mandi Bahauddin (MBD), Jhang (JNG), Khushab (KHS), Okara (OKR), Multan (MLN), Vehari (VHR), Dera Ghazi Khan (DGK), Rajanpur (RJP), Rawalpindi (RWP), Jhelum (JLM), Bahawalpur (BWP), Rahim Yar Khan (RYK). Districts were purposively selected to reflect geographic and socioeconomic diversity across Punjab, including variation in urban–rural composition, population density, income levels, sanitation infrastructure, and access to healthcare services, based on available provincial demographic and public health data. Within each district, a target of approximately 45 febrile children was set to ensure that no single district dominated the overall sample. Eligible children presenting with fever and clinical suspicion of typhoid fever were consecutively invited to participate until district and stratum-specific targets were reached.

### 2.2. Epidemiological Investigations

A structured questionnaire was administered exclusively to parents (mother or father) of the participating children at the time of enrollment. It collected information on demographic characteristics, socioeconomic status, environmental conditions, food and water hygiene practices, awareness of typhoid fever, vaccination history, healthcare-seeking behavior, and prior antibiotic use. Parents served as proxy respondents for both child-related behaviors and their own knowledge, attitudes, and practices that may influence infection risk. Behavioral questions focused on routine, regularly observed household practices, such as handwashing before meals or after toilet use. All data were collected by trained interviewers following a standardized protocol. Prior to data collection, interviewers underwent structured training to ensure consistent administration, neutral questioning, and ethical conduct. They were instructed to consult with the Principal Investigator to clarify any ambiguity in the field, thereby minimizing interviewer-related variability.

The nutritional status of children was assessed using anthropometric measurements. Body weight and height were measured at the time of enrolment using standardized procedures, and body mass index (BMI) was calculated as weight (kg)/height^2^ (m^2^). Nutritional status was classified using WHO age-specific BMI-for-age reference standards. Children with a BMI-for-age Z-score below −2 standard deviations were categorized as underweight or malnourished [15].

Dietary habits were evaluated using a structured questionnaire administered to parents. Parents were asked about the regular consumption of selected food groups by the child, including milk, fruits, protein-rich foods, and street food. “Regular consumption” was operationally defined as intake on at least three days per week.

The handwashing practices were also evaluated. “Regular hand washing” was operationally defined as washing hands with soap and water for 15–20 s before meals and after using the toilet on at least 80% of occasions, as reported by the parents. This definition was applied consistently across all participants to standardize the variable and reduce subjective interpretation.

Typhoid vaccination status was collected via parental report using a structured questionnaire. Due to incomplete or inconsistent record-keeping in healthcare facilities, particularly in rural and nomadic populations, it was not possible to verify vaccination through official records for all children. This self-reported information was used to categorize children as vaccinated or unvaccinated.

### 2.3. Sample Collection and Bacterial Identification

Blood samples were collected for culture from eligible febrile children (≤15 years) who were clinically suspected of having typhoid fever by attending healthcare personnel. Children who had received systemic antibiotics within 72 h prior to sample collection were excluded to reduce the likelihood of false-negative culture results. For blood cultures, briefly, the blood samples (~2 mL) were collected after disinfecting the venipuncture site with 70% isopropyl alcohol and dispensed in a Columbia broth diphasic medium with added SPS (sodium polyanethol sulphonate). The samples were transported to the laboratory and cultured onto MacConkey and blood agar plates. The inoculated plates were incubated in an inverted position at 37 °C for 24 h under aerobic conditions. Colony morphology was examined followed by Gram-staining, and biochemical identification was performed by using the API 20E (bioMérieux, Lyon, France).

### 2.4. Antimicrobial Susceptibility Testing

The antimicrobial susceptibility of *S*. Typhi clinical strains was assessed using the disc diffusion (DD) method by Clinical and Laboratory Standards Institute (CLSI) 2025 guidelines. The antibiotics discs (Oxoid, Hampshire, UK) were used including ampicillin (10 µg), Trimethoprim-sulfamethoxazole (1.25/23.75 µg), chloramphenicol (30 µg), ceftriaxone (30 µg), cefotaxime (30 µg), ciprofloxacin (5 µg), imipenem (10 µg), meropenem (10 µg), doxycycline (30 µg) and azithromycin (15 µg).

The minimum inhibitory concentrations (MICs) of the 10 antibiotics were determined using the broth microdilution method by Clinical and Laboratory Standards institute (CLSI) 2025 guidelines. Twofold serial dilutions of each antibiotic were prepared in cation-adjusted Mueller-Hinton broth. Bacterial suspensions were adjusted to a 0.5 McFarland turbidity standard and further diluted to achieve a final inoculum density of approximately 5 × 10^5^ CFU/mL. following 18 h of incubation at 35 °C, MIC values were recorded as the lowest antibiotic concentrations that completely inhibited visible bacterial growth. For setting quality control ranges, American Type Culture Collection (ATCC) *Escherichia coli* ATCC strain no. 25922 and *P. aeruginosa* ATCC no. 27853 (for carbapenems) were used as a control for the susceptibility testing and determination of MICs. Readings for clinical isolates were taken only when quality control ranges were satisfactory.

Isolates resistant to first-line antibiotics (ampicillin, trimethoprim-sulfamethoxazole, and chloramphenicol) were classified as MDR [16]. Isolates exhibiting additional resistance to fluoroquinolones (ciprofloxacin) and third-generation cephalosporins (ceftriaxone, cefotaxime) were classified as extensively drug-resistant (XDR) [9], in accordance with the definitions of the World Health Organization (WHO) and the Centers for Disease Control and Prevention (CDC).

### 2.5. Genotypic Characterization of Antimicrobial Resistant Determinants

All isolates including susceptible and resistant strains, were screened via PCR to detect antimicrobial resistance genes (ARGs) using specific primers. The ARGs include ESBLs, i.e., *bla*_TEM_, *bla*_SHV_, *bla*_CTX-M-1_ and *bla*_CTX-M-15_ genes [17], Trimethoprim resistance gene encodes *dfrA7* [18], sulfonamide resistance genes including *sul1*, and *sul2*, the *catA1* gene encoding chloramphenicol acetyltransferase [19], and the plasmid-mediated quinolone resistance determinants (*qnrA*, *qnrB*, *qnrS*) [20]. The DNA was extracted through the boiling method. PCR amplification was carried out in a 30 µL reaction volume comprising 15 µL of 2X master mix, 200 nM of each primer, and 1 µL of template DNA, using a T100^TM^ Thermal Cycler (Bio-Rad Laboratories, Inc., Hercules, CA, USA). Positive controls (reference strains with confirmed ARGs) and negative controls (no-template DNA) were included in each PCR run to validate amplification specificity. Following amplification, 5 µL of each PCR product was separated by 1.2% agarose gel electrophoresis at 120 V in 1X TAE buffer for 35 min and visualized with a UV transilluminator.

### 2.6. Statistical Analysis

The data were analyzed using Microsoft Excel and Statistical Package for Social Science (SPSS) software, version 27. Categorical data are presented as frequencies and percentages. Geographic distribution of antimicrobial resistance patterns was analyzed using chi-square test for independence. Proportions are reported with 95% confidence intervals calculated using the Wilson score method. Statistical significance was determined with a threshold *p*-value of ≤0.05.

## 3. Results

### 3.1. Demographic and Socioeconomic Characteristics of Participants

Of 900 febrile children enrolled, 374 (41.5%) were confirmed positive for *S.* Typhi by blood culture. Demographic analysis revealed no significant association between gender and infection status, with males comprising 52.1% (195/374) of positive cases and females 46% (174/374) (*p* = 0.693). Similarly, the distribution of *S.* Typhi infection across age groups was not statistically significant (*p* = 0.752). The largest proportion of participants (318/900, 35.3%) were aged 6–12 years, of whom 130 (34.8%) were infected. The 1–5 years age group comprised 255/900 (28.3%) of participants, with 113 (30.2%) infected cases.

*S.* Typhi positivity rates varied geographically across 20 districts though this variation did not reach statistical significance (*p* = 0.083). In contrast, a highly significant association was observed between the area of residence and *S.* Typhi infection (*p* < 0.0001). Participants were equally distributed across urban, rural, and nomadic areas (300 each) However, nomadic communities bore the highest burden, accounting for 48.1% (180/374) of all positive cases. Urban areas contributed 125 cases (33.4%), while rural areas had the lowest proportion of infections at 18.4% (69/374).

Family income also demonstrated a strong inverse correlation with infection risk (*p* < 0.0001). A large proportion of participants (395/900, 43.9%) were from low-income households (earning < PKR 20,000 per month); notably, this group accounted for 67.1% (251/374) of all *S.* Typhi cases. Middle-income households (PKR 20,000–50,000/month; 315/900, 35% of participants) accounted for 23.5% (88/374) of cases, while high-income households (>PKR 50,000/month; 190/900, 21.1% of participants) represented only 9.4% (35/374) of infections (Table 1).

### 3.2. Nutritional, Behavioral and Environmental Risk Factors for Salmonella Typhi

Our analysis identified several environmental, nutritional, and behavioral factors significantly associated with *S.* Typhi infection. Exposure to crowded areas was reported for 64.8% (583/900) of children and was significantly associated with infection, as 71.1% (266/374) of positive cases came from this group (*p* = 0.001). The strongest environmental predictor was the presence of stagnant water; this was reported by 530 participants, and it accounted for 82.4% (308/374) of all positive cases (*p* < 0.001).

Nutritional status and dietary habits were also major determinants. BMI-defined malnutrition, observed in 52% (468/900) of children, was significantly associated with infection (57.5% of positive cases, *p* = 0.0067). Conversely, a protective effect was demonstrated by the regular consumption of milk, fruits, and protein-rich foods. This was reported by 42.1% (379/900) of participants and was associated with a significantly lower proportion of infections (32.6% of positive cases, *p* = 0.0001). Frequent street food consumption was common (53.9%, 485/900) but was not significantly associated with infection risk (*p* = 0.746).

Several hygiene and awareness practices were strongly protective. Regular handwashing before meals (reported by 36.2%, 326/900) and after using the toilet (reported by 45%, 405/900) were both associated with a significantly lower risk of infection (22.5% and 31.8% of positive cases, respectively; *p* < 0.0001 for both). Similarly, awareness of typhoid transmission (29%, 263/900) was linked to a much lower infection rate (15% of positives, *p* < 0.0001). The practice of washing raw vegetables (25.7%, 231/900) was also significantly protective (20.1% of positives, *p* < 0.0001). In contrast, drinking water from educational institutions (37.9%, 341/900) was a significant risk factor, associated with 55.9% of positive cases (*p* < 0.0001).

Vaccination and antibiotic use patterns were critically important. Receipt of the typhoid conjugate vaccine (TCV) was reported for 33.8% (304/900) of children and demonstrated a strong protective effect, with only 16.8% (63/374) of positive cases occurring in this group (*p* < 0.0001). Concerningly, antibiotic misuse was prevalent and linked to higher infection rates. Use of antibiotics without a prescription upon symptom onset (17.9%, 161/900) and use based on a previous prescription (41.5%, 374/900) were both significantly associated with infection (*p* < 0.0001). In contrast, completing the full course of prescribed antibiotics (29.6%, 266/900) was strongly protective (13.6% of positives, *p* < 0.0001). Awareness of antibiotic resistance itself (parents/caregivers) was also a significant protective factor (*p* < 0.0001) (Table 2).

### 3.3. Antimicrobial Resistance Profile

**Figure 1:**
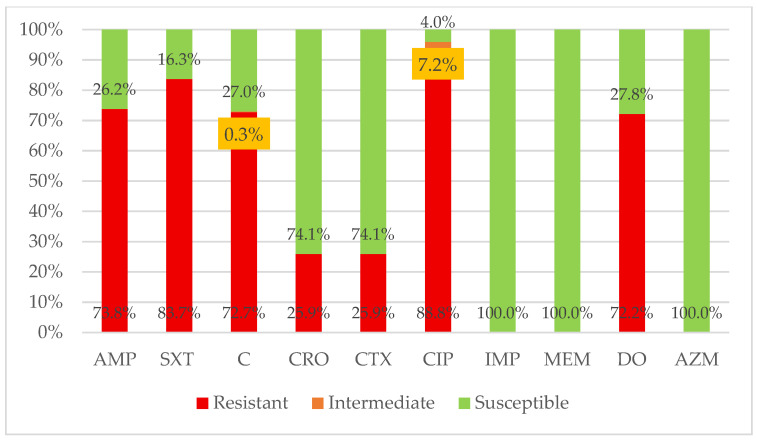
Antimicrobial susceptibility pattern of *Salmonella* Typhi isolates against commonly used antibiotics. AMP: Ampicillin; SXT: trimethoprim–sulfamethoxazole; C: chloramphenicol; CRO: ceftriaxone; CTX: cefotaxime; CIP: ciprofloxacin; IMP: imipenem; MEM: meropenem; DO: doxycycline; AZM: azithromycin.

The antimicrobial susceptibility testing of *S.* Typhi isolates revealed a high prevalence of resistance to first line and second-line antibiotics. Resistance rates were alarmingly high to quinolones (ciprofloxacin, 88.8%) and first-line agents including sulfonamides (trimethoprim-sulfamethoxazole, 83.7%), penicillin (ampicillin, 73.8%), and amphenicols (chloramphenicol, 72.7%). Resistance to tetracycline (doxycycline) was 72.2%. Notably, 25.9% of isolates were resistant to third-generation cephalosporins (ceftriaxone and cefotaxime), underscoring the spread of difficult-to-treat strains. In contrast, all isolates remained fully susceptible to carbapenems (imipenem, meropenem) and macrolides (azithromycin), confirming these as the last-line effective therapeutic options in this region (Figure 1).

**Figure 2:**
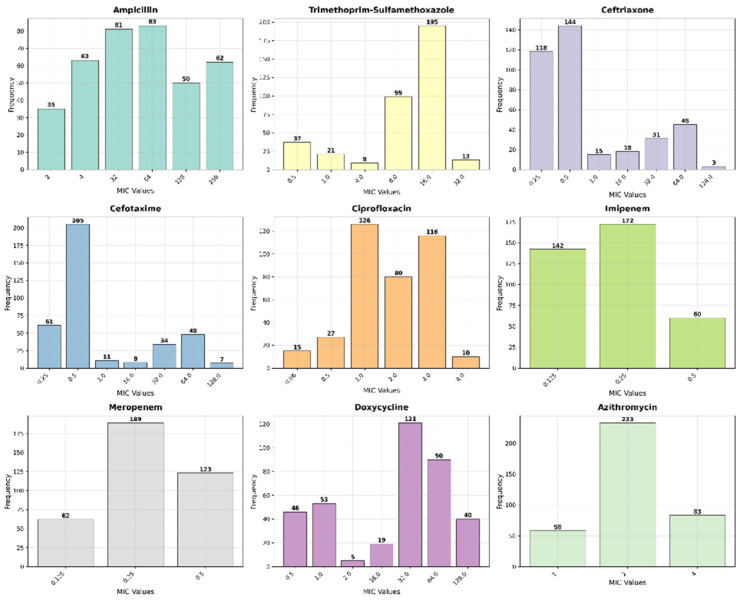
MIC Distribution of different antimicrobial agents against *Salmonella* Typhi isolates.

**Figure 3:**
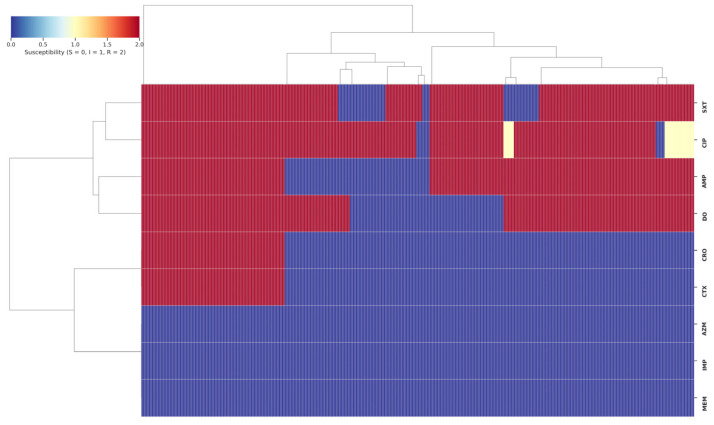
Heatmap representing the antimicrobial susceptibility profiles of *Salmonella* Typhi isolates. Each column corresponds to an individual isolate, and each row represents an antibiotic tested. The color gradient indicates susceptibility status: blue for susceptible (S), yellow for intermediate (I), and red for resistant (R).

Analysis of Minimum Inhibitory Concentrations (MICs) provided further resolution of the resistance landscape. For ampicillin, a substantial proportion of resistant isolates exhibited high MIC values (≥32 μg/mL), with 16.5% (*n* = 62) reaching ≥256 μg/mL. Similarly, high-level resistance was common for ceftriaxone and cefotaxime, with 12% (*n* = 45) of resistant isolates for each agent exhibiting MICs of 64 μg/mL. The high rate of ciprofloxacin resistance was reflected in MICs of 4 μg/mL for 31% (*n* = 116) of isolates. All isolates were confirmed to be susceptible to carbapenems (MICs ≤ 0.25 μg/mL) and azithromycin (Figure 2 and Figure 3).

### 3.4. Antimicrobial Resistance Determinants

Genotypic analysis revealed a strong concordance between phenotypic resistance profiles and the underlying genetic determinants. The high prevalence of resistance to first-line antibiotics was explained by the frequent detection of corresponding resistance genes: *sul1* (83.7%), *sul2* (79.7%), and *dfrA7* (81.3%) for trimethoprim-sulfamethoxazole; *catA1* (64.9%) for chloramphenicol; and *bla*_TEM_ (60.5%) for ampicillin. The absence of the SHV gene indicates it does not contribute to resistance in this population.

**Figure 4:**
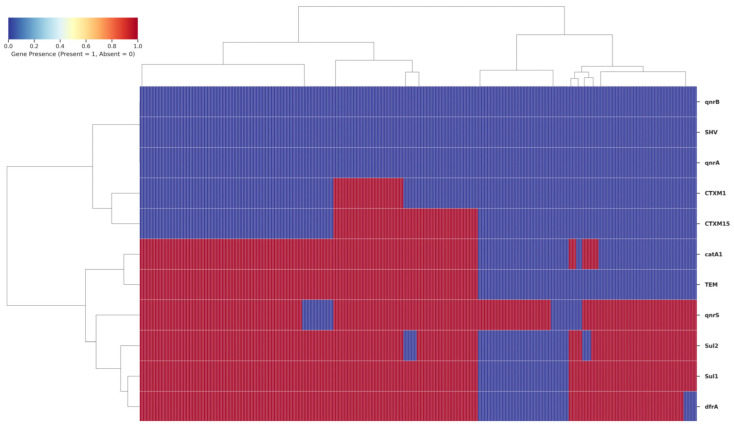
Heatmap illustrating the distribution of antimicrobial resistance genes among *Salmonella* Typhi isolates. Each column represents an isolate, and each row denotes a specific resistance gene. The color gradient indicates gene presence (red) or absence (blue). Hierarchical clustering demonstrates the co-occurrence of multiple resistance determinants, including β-lactamase (*bla*_TEM_, *bla*_CTXM_), sulfonamide (*sul1*, *sul2*), and quinolone (*qnrA*) resistance genes, suggesting the emergence of multidrug-resistant genetic profiles among isolates.

For broader-spectrum agents, the presence of extended-spectrum β-lactamase (ESBL) genes *bla*_CTX-M-1_ (12.5%) and *bla*_CTX-M-15_ (25.9%) correlated with observed resistance to third-generation cephalosporins. The exceptionally high rate of ciprofloxacin resistance (88%) was strongly associated with the pervasive presence of the *qnrS* gene (88.8%), which was the sole plasmid-mediated quinolone resistance determinant identified, as qnrA and qnrB were absent (Figure 4).

**Figure 5:**
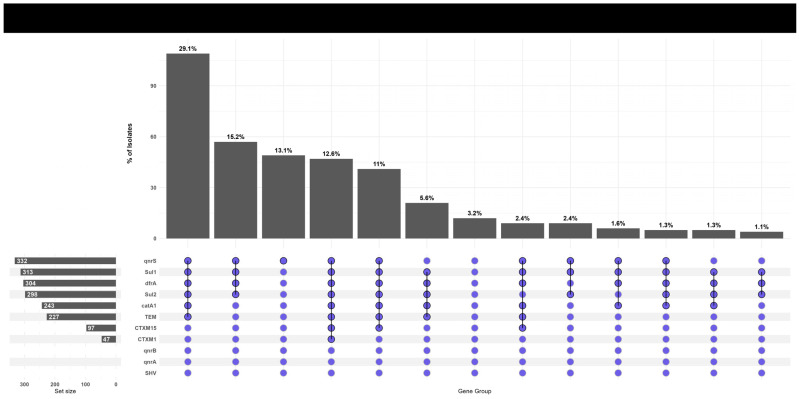
UpSet plot illustrating the co-occurrence patterns of antimicrobial resistance genes among *Salmonella* Typhi isolates. Each vertical bar represents a unique combination of genes, and the bar height indicates the proportion of isolates carrying that gene combination. The connected blue dots denote co-existing genes within each group.

The co-occurrence patterns of antimicrobial resistance genes among *Salmonella* Typhi isolates are illustrated in Figure 5. The UpSet plot depicts the gene combinations and their relative frequencies, highlighting the extent of concurrent resistance determinants within the analyzed isolates.

### 3.5. Distribution of MDR and XDR S. Typhi

Analysis of 374 isolates revealed a significant association between geographic area and antimicrobial resistance profiles (*p* < 0.001) (Table 3). The burden of extensively drug-resistant (XDR) *S.* Typhi was disproportionately high in urban areas, which accounted for 60.8% (95% CI: 50.4–70.5%) of all XDR isolates. This burden is approximately three-fold higher than in rural (20.6%; 95% CI: 13.3–30.0%) or nomadic (18.6%; 95% CI: 11.6–27.6%) populations. In contrast, nomadic populations exhibited the highest proportion of non-multidrug-resistant (non-MDR) isolates (68.7%; 95% CI: 60.0–76.5%), compared to urban (19.0%; 95% CI: 12.9–26.9%) and rural (12.2%; 95% CI: 7.3–19.0%) areas. Multidrug-resistant (MDR) isolates were most prevalent among nomadic populations (49.3%; 95% CI: 41.0–57.7%), compared to urban (28.1%; 95% CI: 21.1–36.1%) and rural (22.6%; 95% CI: 16.2–30.2%) settings.

## 4. Discussion

This comprehensive surveillance study elucidates the complex interplay of geographic, socioeconomic, and behavioral factors driving the high burden of typhoid fever and antimicrobial resistance among febrile children in Punjab, Pakistan. Our findings, that 41.5% of children presented with fever were culture-positive for *S.* Typhi underscores the persistent and severe burden of this disease in the region. The epidemiology is not uniform; it is sharply defined by disparities in income, residence, and hygiene practices, and is further compounded by an alarming prevalence of multidrug-resistant (MDR) and extensively drug-resistant (XDR) strains.

Consistent with prior reports, we found a slightly higher incidence in males and in children aged 6–12 years, a demographic frequently engaged in activities outside the home that may increase exposure risk [21,22]. However, the most powerful predictors were socioeconomic. The overwhelming majority of cases (67.1%) originated from low-income households, reinforcing the well-established link between poverty, inadequate water, and poor sanitation in typhoid transmission [12]. While district-level heterogeneity was observed, the lack of statistical significance suggests that hyper-local environmental conditions, rather than administrative boundaries, are the primary determinants of transmission risk. Environmental, educational, and behavioral factors likely vary across districts and may influence the risk of infection, potentially introducing bias in district-specific estimates. While our study aimed to capture geographic and socioeconomic diversity, the small per-district sample and facility-based recruitment limit precise district-level inference. Therefore, broader patterns by urban, rural, and nomadic populations, and by socioeconomic status, may provide more reliable insights into risk determinants.

Our data provides robust evidence for the role of specific environmental and behavioral risk factors. Exposure to stagnant water was the strongest environmental predictor of infection, while crowded living conditions significantly increased risk. Malnutrition markedly heightened susceptibility, whereas a diet rich in milk, fruits, and protein was strongly protective, highlighting the critical role of nutrition in immune defense [23,24]. Key hygiene practices, particularly handwashing before meals and after using the toilet, were associated with significantly reduced risk. Conversely, drinking water from educational institutions emerged as a major risk factor, pointing to a specific and urgent public health intervention point. The protective effect of typhoid conjugate vaccine (TCV) underscores its vital role in control strategies [25]. However, this benefit is threatened by pervasive antibiotic misuse, which was reported in most cases. Completing a full prescribed antibiotic course was protective [26], while inappropriate use directly fuels the antimicrobial resistance crisis, a phenomenon whose distinct geographic and genetic architecture is revealed in the following findings.

Driven in part by this pervasive antibiotic misuse, our study reveals significant geographic variation in antimicrobial resistance patterns of typhoid fever, with extensively drug-resistant (XDR) *S.* Typhi concentrated primarily in urban areas—likely due to greater antibiotic access increasing selection pressure, higher population density facilitating transmission, and healthcare facilities serving as amplification sites—while nomadic populations show a predominance of non-multidrug-resistant (Non-MDR) strains, possibly reflecting geographic isolation, limited antibiotic exposure, or maintenance of susceptible strain reservoirs. In contrast, multidrug-resistant (MDR) isolates are relatively uniformly distributed across all geographic areas, suggesting these strains are well-established throughout the region following their longer history in South Asia. These findings have critical implications for public health strategy: urban areas require enhanced surveillance and tailored empirical treatment protocols accounting for high XDR prevalence, nomadic populations need improved healthcare access balanced with antimicrobial stewardship to prevent resistance emergence, and aggregated national data may mask important sub-population variations that could lead to inappropriate treatment choices. The study underscores the necessity of localized surveillance systems and geographically tailored treatment algorithms to optimize patient outcomes while minimizing further resistance development across diverse population settings.

*sul1* (83.7%), *sul2* (79.7%), followed by *dfrA7* (81.3%), *catA1* (64.9%) and *bla*_TEM_ (60.5%), *bla*_CTX-M-1_ (12.5%), *bla*_CTX-M-15_ (25.9%) and *qnrS* (88.8%),

These distinct geographic patterns of resistance are underpinned by specific genetic determinants. The concordance we found between phenotypic resistance and its underlying genetic basis confirms the molecular mechanisms driving the MDR and XDR epidemic. In this study we found a strong concordance between phenotypic resistance and the underlying genetic determinants, confirming the molecular mechanisms driving the MDR and XDR epidemic. The high prevalence of *sul1* (83.7%), *sul2* (79.7%), *dfrA7* (81.3%), *catA1* (64.9%), and *bla*_TEM_ (60.5%) genes explain the widespread resistance to first-line antibiotics. These patterns are broadly consistent with molecular surveys from Pakistan and other countries that identify the same mobile resistance determinants as dominant drivers of first line drug resistance in *S.* Typhi [2,9,27]. The detection of *bla*_CTX-M_ family variants (*bla*_CTX-M-1_,12.5% and *bla*_CTX-M-15_, 25.9%) in our isolates echoes reports from Pakistan documenting *bla*_CTX-M_-mediated ESBL activity in third-generation cephalosporin resistant *S.* Typhi. Notably, *bla*_CTX-M-15_ has been repeatedly implicated in XDR clones identified in urban centers such as Karachi [28].

Our findings underscore the urgent need for integrated, multi-pronged public health strategies tailored to the distinct vulnerabilities of urban, rural, and nomadic populations in Punjab. A proactive approach must be prioritized, beginning with the rapid scale-up of typhoid conjugate vaccine (TCV) immunization, particularly among high-burden nomadic and low-income communities. This must be coupled with sustained investments in water and sanitation infrastructure to ensure access to safe drinking water, with specific attention to mitigating the identified risk posed by water sources in educational institutions. Concurrently, widespread public health education campaigns are essential to promote essential hygiene practices such as handwashing, improving nutritional status, and combat the dangerous misuse of antibiotics through effective stewardship. Finally, the strong genotype phenotype correlation we observed mandates the implementation of robust molecular surveillance systems to monitor the evolution and spread of resistant clones, enabling preemptive containment. Together, these integrated, population-tailored interventions are critical for mitigating the dual burden of typhoid and antimicrobial resistance in Punjab; the lessons learned here have direct relevance for national policy in Pakistan and for global efforts to combat drug-resistant enteric fever in endemic settings.

This study has several limitations. Recruitment was facility-based and employed a non-random sampling approach among febrile, clinically suspected children, which may limit the generalizability of the findings beyond healthcare-seeking populations and precludes inference to population-level prevalence. The nutritional status was assessed using objective BMI measurements, dietary intake data were based on parental self-report and may be subject to recall bias. Handwashing practices were based on parental self-report and may be subject to social desirability or recall bias; however, the use of a standardized operational definition moderates some variability. Vaccination status was based on parental report rather than documented records, which may lead to misclassification or recall bias. However, this approach was necessary given the incomplete and inconsistent vaccination records in the study settings. Further, the sample size per district was limited, potentially constraining the generalizability of district-specific estimates. The molecular analysis was confined to a panel of known resistance genes; whole-genome sequencing would provide a more comprehensive view of resistance mechanisms, plasmid dynamics, and phylogenetic relationships. Finally, the cross-sectional design identifies associations but cannot establish causal relationships between risk factors and infection.

## 5. Conclusions

This extensive surveillance study reveals that the persistently high burden of typhoid fever and antimicrobial resistance (AMR) among children in Punjab, Pakistan, is critically defined by geographic residence, socioeconomic status, and behavioral factors. The alarming prevalence of resistance, particularly to first-line drugs and fluoroquinolones, presents a formidable challenge to clinical management. Our findings, which demonstrate distinct AMR patterns across urban, rural, and nomadic populations, mandate a comprehensive and geographically tailored control strategy. Priority must be given to expanding typhoid conjugate vaccine (TCV) coverage in all high-burden communities. Concurrently, strengthening water, sanitation, and hygiene (WASH) infrastructure with urgent attention to risks like unsafe water in schools is crucial to disrupt transmission. Public health campaigns must promote essential hygiene and nutrition. Furthermore, enforcing effective antibiotic stewardship through regulation of over-the-counter sales and adherence to evidence-based guidelines is imperative to curb the rise in resistant strains. The integration of molecular AMR surveillance will be vital for monitoring resistance evolution and guiding therapy. Together, these integrated interventions are essential to mitigate the dual threat of typhoid fever and AMR in this vulnerable pediatric population.

## Figures and Tables

**Table 1:** Distribution of demographic and socioeconomic variables among study participants (*n* = 900).

Variable	Category	Total *n* (%)	*S.* Typhi Positive (*n* = 374)	*S*. Typhi Negative (*n* = 526)	*p*-Value
Gender	Male	471 (52.3%)	195 (52.1%)	276 (52.5%)	0.693
	Female	420 (46.7%)	174 (46.5%)	246 (46.8%)	
	Transgender	9 (1.0%)	5 (1.3%)	4 (0.8%)	
Age Group	0–12 months	68 (7.6%)	28 (7.5%)	40 (7.6%)	0.752
	1–5 years	255 (28.3%)	113 (30.2%)	142 (27.0%)	
	6–12 years	318 (35.3%)	130 (34.8%)	188 (35.7%)	
	13–15 years	259 (28.8%)	103 (27.5%)	156 (29.7%)	
Area	Urban	300 (33.3%)	125 (33.4%)	175 (33.3%)	<0.0001
	Rural	300 (33.3%)	69 (18.4%)	231 (43.9%)	
	Nomadic	300 (33.3%)	180 (48.1%)	120 (22.8%)	
Monthly Income (PKR)	<20,000	395 (43.9%)	251 (67.1%)	144 (27.4%)	<0.0001
	20,000–50,000	315 (35.0%)	88 (23.5%)	227 (43.1%)	
	>50,000	190 (21.1%)	35 (9.4%)	155 (29.5%)	
Districts	Lahore	45 (5.0%)	21 (5.6%)	24 (4.6%)	0.083
	Faisalabad	45 (5.0%)	22 (5.9%)	23 (4.4%)	
	Sheikhupura	45 (5.0%)	21 (5.6%)	24 (4.6%)	
	Gujranwala	45 (5.0%)	16 (4.3%)	29 (5.5%)	
	Narowal	45 (5.0%)	25 (6.7%)	20 (3.8%)	
	Sargodha	45 (5.0%)	21 (5.6%)	24 (4.6%)	
	Sahiwal	45 (5.0%)	14 (3.7%)	31 (5.9%)	
	Gujrat	45 (5.0%)	20 (5.3%)	25 (4.8%)	
	Mandi Bahauddin	45 (5.0%)	14 (3.7%)	31 (5.9%)	
	Jhang	45 (5.0%)	12 (3.2%)	33 (6.3%)	
	Khushab	45 (5.0%)	18 (4.8%)	27 (5.1%)	
	Okara	45 (5.0%)	14 (3.7%)	31 (5.9%)	
	Multan	45 (5.0%)	20 (5.3%)	25 (4.8%)	
	Vehari	45 (5.0%)	26 (7.0%)	19 (3.6%)	
	Dera Ghazi Khan	45 (5.0%)	18 (4.8%)	27 (5.1%)	
	Rajanpur	45 (5.0%)	19 (5.1%)	26 (4.9%)	
	Rawalpindi	45 (5.0%)	24 (6.4%)	21 (4.0%)	
	Jhelum	45 (5.0%)	17 (4.5%)	28 (5.3%)	
	Bahawalpur	45 (5.0%)	13 (3.5%)	32 (6.1%)	
	Rahim Yar Khan	45 (5.0%)	19 (5.1%)	26 (4.9%)	

**Table 2:** Comparison of Nutritional, Behavioral and Environmental Risk Factors between *S*. Typhi positive and negative participants.

Variable	Category	Total *n* = 900 (%)	*S*. Typhi Positive (*n* = 374)	*S*. Typhi Negative (*n* = 526)	*p*-Value
1. Visits crowded areas	Yes	583 (64.8%)	266 (71.1%)	317 (60.3%)	0.001
	No	317 (35.2%)	108 (28.9%)	209 (39.7%)	
2. Foul-smelling or stagnant water in surroundings	Yes	530 (58.9%)	308 (82.4%)	222 (42.2%)	<0.0001
	No	370 (41.1%)	66 (17.6%)	304 (57.8%)	
3. Underweight or malnourished (BMI-based)	Yes	468 (52.0%)	215 (57.5%)	253 (48.1%)	0.0067
	No	432 (48.0%)	159 (42.5%)	273 (51.9%)	
4. Consumes milk, fruits, or proteins (meat/eggs) regularly	Yes	379 (42.1%)	122 (32.6%)	257 (48.9%)	<0.0001
	No	521 (57.9%)	252 (67.4%)	269 (51.1%)	
5. Eats street food or snacks	Often	485 (53.9%)	201 (53.7%)	284 (54.0%)	0.746
	Occasionally	256 (28.4%)	103 (27.5%)	153 (29.1%)	
	Never	159 (17.7%)	70 (18.7%)	89 (16.9%)	
6. Uses unpasteurized dairy products	Never	194 (21.6%)	64 (17.1%)	130 (24.7%)	<0.0001
	Sometimes	262 (29.1%)	77 (20.6%)	185 (35.2%)	
	Often	358 (39.8%)	178 (47.6%)	180 (34.2%)	
	Always	86 (9.6%)	55 (14.7%)	31 (5.9%)	
7. Wash raw vegetables or fruits	Never	275 (30.6%)	147 (39.3%)	128 (24.3%)	<0.0001
	Sometimes	241 (26.8%)	92 (24.6%)	149 (28.3%)	
	Often	153 (17.0%)	60 (16.0%)	93 (17.7%)	
	Always	231 (25.7%)	75 (20.1%)	156 (29.7%)	
8. Knowledge about typhoid transmission	Yes	263 (29.2%)	56 (15.0%)	207 (39.4%)	<0.0001
	No	637 (70.8%)	318 (85.0%)	319 (60.6%)	
9. Drinks water from educational institution	Never	97 (10.8%)	23 (6.1%)	74 (14.1%)	<0.0001
	Sometimes	239 (26.6%)	67 (17.9%)	172 (32.7%)	
	Often	223 (24.8%)	75 (20.1%)	148 (28.1%)	
	Always	341 (37.9%)	209 (55.9%)	132 (25.1%)	
10. Washes hands before eating	Never	97 (10.8%)	70 (18.7%)	27 (5.1%)	<0.0001
	Sometimes	317 (35.2%)	159 (42.5%)	158 (30.0%)	
	Often	160 (17.8%)	61 (16.3%)	99 (18.8%)	
	Always	326 (36.2%)	84 (22.5%)	242 (46.0%)	
11. Washes hands after using the toilet	Never	119 (13.2%)	80 (21.4%)	39 (7.4%)	<0.0001
	Sometimes	227 (25.2%)	136 (36.4%)	91 (17.3%)	
	Often	149 (16.6%)	39 (10.4%)	110 (20.9%)	
	Always	405 (45.0%)	119 (31.8%)	286 (54.4%)	
12. Received the typhoid conjugate vaccine (TCV)	Yes	304 (33.8%)	63 (16.8%)	241 (45.8%)	<0.0001
	No	218 (24.2%)	102 (27.3%)	116 (22.1%)	
	Don’t know	378 (42.0%)	209 (55.9%)	169 (32.1%)	
13. Antibiotics without a doctor’s prescription	Yes, when I notice symptoms	161 (17.9%)	69 (18.4%)	92 (17.5%)	<0.0001
	Yes, based on past prescriptions	374 (41.5%)	219 (58.6%)	155 (29.5%)	
	No, I never self-medicate	300 (33.3%)	57 (15.2%)	243 (46.2%)	
	Not sure	65 (7.2%)	29 (7.8%)	36 (6.8%)	
14. Completes full course of antibiotics prescribed	Always	266 (29.6%)	51 (13.6%)	215 (40.9%)	<0.0001
	Most of the time	133 (14.8%)	29 (7.8%)	104 (19.8%)	
	Sometimes	203 (22.6%)	95 (25.4%)	108 (20.5%)	
	Rarely	104 (11.6%)	60 (16.0%)	44 (8.4%)	
	Never	194 (21.5%)	139 (37.2%)	55 (10.5%)	
15. Knowledge about antibiotic resistance	Bacteria’s ability to survive antibiotics	91 (10.1%)	24 (6.4%)	67 (12.7%)	<0.0001
	Increased risk of treatment failure	62 (6.9%)	8 (2.1%)	54 (10.3%)	
	Overuse/misuse of antibiotics	164 (18.2%)	19 (5.1%)	145 (27.6%)	
	All of the above	98 (10.9%)	28 (7.5%)	70 (13.3%)	
	None of the above	485 (53.9%)	295 (78.9%)	190 (36.1%)	

**Table 3:** Association Between Geographic Area and Antimicrobial Resistance Patterns (Non-MDR, MDR, XDR) in *S.* Typhi Isolates.

Area	Non-MDR *n* (%) (95% CI)	MDR *n* (%) (95% CI)	XDR *n* (%) (95% CI)	*p*-Value *
Urban	25 (19.0%) (12.9–26.9)	41 (28.1%) (21.1–36.1)	59 (60.8%) (50.4–70.5)	<0.001
Rural	16 (12.2%) (7.3–19.0)	33 (22.6%) (16.2–30.2)	20 (20.6%) (13.3–30.0)	
Nomadic	90 (68.7%) (60.0–76.5)	72 (49.3%) (41.0–57.7)	18 (18.6%) (11.6–27.6)	
Total	131 (100%)	146 (100%)	97 (100%)	—

* Chi-square test for independence; CI = Confidence Interval calculated using Wilson score method for proportions.

## Data Availability

The data presented in this study are available on request from the corresponding author. The data are not publicly available due to privacy and ethical restrictions.
